# Analysis of Angiotensin Converting Enzyme, Endothelial Nitric Oxide Synthase & Serotonin Gene Polymorphisms among Atrial Septal Defect Subjects with and without Pulmonary Arterial Hypertension

**DOI:** 10.3390/jcdd5030048

**Published:** 2018-09-18

**Authors:** Nur Ilyana Jaafar, Ramachandran Vasudevan, Patimah Ismail, Ahmad Fazli Abdul Aziz, Nur Afiqah Mohamad, Geetha Kandavello, Raja Nurzatul Effah Raja Adnan, Vinod Balasubramaniam

**Affiliations:** 1 Department of Biomedical Sciences, Faculty of Medicine and Health Sciences, Universiti Putra Malaysia, Serdang 43400, Malaysia; elle.ilyana@gmail.com; 2 Malaysian Research Institute on Ageing, Universiti Putra Malaysia, Serdang 43400, Malaysia; nur_iqa87@yahoo.com (N.A.M.); r_zatulefa@upm.edu.my (R.N.E.R.A.); 3 Department of Medicine, Faculty of Medicine and Health Sciences, Universiti Putra Malaysia, Serdang 43400, Malaysia; afazli@upm.edu.my; 4 Department of Pediatric Clinic, National Heart Institute, Jalan Tun Razak, Kuala Lumpur 50400, Malaysia; geetha@ijn.com.my; 5 Jeffrey Cheah School of Medicine and Health Sciences, Monash University Malaysia, Bandar Sunway 47500, Malaysia; vinod.balasubramaniam@monash.edu

**Keywords:** pulmonary artery hypertension, atrial septal defect, genetic polymorphisms

## Abstract

Genetic polymorphisms are variations in DNA sequences which can influence either disease susceptibility, severity, or prognosis. Pulmonary arterial hypertension (PAH) is one of the complications that occurs in certain patients who have atrial septal defect (ASD). This study seeks to determine the association of gene polymorphisms with the pathogenesis of PAH in ASD patients. This study was conducted on 30 ASD patients with PAH, and 50 ASD patients who were not diagnosed with PAH. All respondents were Malay. Patients were selected based on stringent inclusion and exclusion criteria. Molecular analyses were done to detect the genetic polymorphisms of angiotensin converting enzyme (*ACE I/D*), serotonin transporter (*5-HTTLPR*), endothelial nitric oxide synthase (*eNOS*) *G894T*, and *eNOS 4b/4a*. The genotypes of these genetic polymorphisms were determined using conventional PCR and PCR-RFLP methods. The PCR products were analysed using agarose gel electrophoresis. Statistical analysis was done using SPSS Version 22. Clinical characteristics, such as the diameter of ASD, mean arterial pressure (MAP), and mean pulmonary artery pressure (mPAP) differed significantly (*p* < 0.05). Based on the statistical analysis, *ACE I/D*, *eNOS G894T*, and *eNOS 4b/4a* do not contribute to the progression of PAH amongst ASD patients (*p* > 0.05). However, the L allele of the *5-HTTLPR* gene polymorphism may have an affect on the development of PAH in ASD patients (*p* < 0.05).

## 1. Introduction

Atrial septal defect (ASD) is a congenital heart defect caused by the presence of an interatrial communication. ASD is usually asymptomatic in children, but in adults, it may be complicated by pulmonary arterial hypertension (PAH). Since PAH patients do not show obvious symptoms at the early stage, there may be delayed diagnosis and treatment [1]. PAH, a disease that affects small pulmonary arteries, is characterised by an increase in the pressure of pulmonary artery and pulmonary vessels’ resistance, which can lead to death due to right ventricular failure [2]. The normal blood pressure of a pulmonary artery at rest is usually around 8 mmHg to 20 mmHg, but in pulmonary arterial hypertensive individuals, the mean pulmonary arterial pressure at rest is >25 mmHg with normal pulmonary capillary wedge pressure [3]. In most cases, ASD causes left-to-right shunts [4]. The direction and magnitude of the shunting strongly depends on the size of the defect and the relative compliance of the left and right that can change over time [2]. The incidence of PAH is around 2 cases per a million population per year, and can affect individuals at any age, but is most common among adults between 36 to 50 years of age [1]. Patients with ASD are usually asymptomatic and tend to develop PAH after turning 30. However, in some patients, the onset of PAH is noted earlier, and even in small ASD with insignificant left to right shunts. The reason remains unclear. It is believed that in this group of patients, the pathophysiology of PAH is more similar to those with idiopathic PAH.

There are several genes being hypothesised to be involved in PAH, such as serotonin (5-HT), endothelial nitric oxide synthase (*eNOS*), and angiotensin converting enzyme (*ACE I/D*) [5,6]. A conducted meta-analysis showed a significant association between serotonin transporter (SERT) polymorphism with idiopathic pulmonary hypertension [7], but there is a lack of study on SERT polymorphism with PAH in ASD. A functional gene polymorphism has been identified at the promoter region of the 5-HT gene, a 44 bp insertion/deletion polymorphism (*5-HTTLPR*) [8]. A study in pediatric patients of idiopathic PAH shows the presence of homozygous L genotype in 90% of the patients, suggesting the role of 5-HTT in the development of pulmonary hypertension both in children and adults [9].

Nitric oxide synthase is one of the candidate genes involved in PAH. The *NOS3* or *eNOS* gene that encodes eNOS is expressed by vascular endothelial cells. Variations in the gene will alter nitric oxide (NO) synthesis. Polymorphisms of the *eNOS* gene have been studied in several populations in relation to IPAH [10,11] and cardiovascular disorders [12].

The role of *ACE* insertion/deletion (I/D) polymorphism on the development of PAH will be investigated in this study as well. The *ACE* gene, a major component in renin angiotensin aldosterone system, has been implicated in the pathogenesis of cardiovascular disease [13] and essential hypertension [14]. I/D polymorphism of *ACE* gene is one of the most common polymorphisms being studied [15,16]. The risk of developing coronary artery disease, myocardial infarction, or left ventricular hypertrophy increase with the presence of the *ACE* DD genotype [17,18]. Therefore, it has been postulated that the *ACE* genotype plays a role in PAH progression by modulating the expression of the *ACE* gene [19].

Since genetic diversity exists among different ethnic populations, and taking into account the fact that the association in one population could not be extrapolated to another, this study only involves Malay patients. In Malaysia, there is lack of data on the association between genetic polymorphisms with PAH and ASD. This stimulated us to study genetic polymorphisms in relation to the early progression of PAH associated with ASD.

## 2. Materials and Methods

This study comprises of 30 ASD subjects with pulmonary arterial hypertension (cases), and 50 ASD subjects without pulmonary arterial hypertension (controls). The subjects were recruited from the Pediatric Clinic of the National Heart Institute. Sample size was calculated by aiming at 80% statistical power. Prior to recruiting the subjects, ethical approval was obtained from the Ethic Institutional Review Board of Universiti Putra Malaysia (UPM) and the Ethics Committee of the National Heart Institute. The work was carried out in accordance with The Code of Ethics of The World Medical Association (Declaration of Helsinki) for experiments on humans. Consent was obtained from all subjects before recruiting them, and the socio-demographic and medical history of each subject was recorded. Subjects were diagnosed with established PAH by right heart catherisation and with secundum Atrial Septal Defect, with no other cardiac anomaly. The next criteria are subjects with mean pulmonary arterial pressure >50% mean systemic pressure, and mean pulmonary capillary wedge pressure <2 kPa (15 mmHg). Patients who are genotypically diagnosed with Down Syndrome with abnormally accelerated PAH or PAH related to other etiologies and patients suffering from other than secundum ASD were excluded from the study.

### 2.1. Genetic Analysis

A total of 4 mL of blood was collected from subjects’ peripheral blood leukocytes into an EDTA tube (Becton Dickinson, East Rutherford, NJ, USA) by a qualified phlebotomist. Plasma was separated from the blood by centrifugation and stored at −20 °C for further analysis. Genomic DNA from peripheral blood was isolated by using the QIAamp Blood DNA Mini Kit (QIAGEN, Hilden, Germany). The purity of extracted DNA was checked by using a Biophotometer (Eppendorf, Hamburg, Germany). Each gene polymorphism underwent PCR by using their respective primers and specific reaction conditions (Table 1).

The PCR reactions were done by using a G-Storm GS1 Thermal Cyclers (Somertone Biotechnology Center, Somerset, UK). The PCR products were then kept at −20 °C for further analysis. The PCR products were determined by using agarose gel electrophoresis, and visualised under UV light. The results were validated by choosing 20% of samples at random, and a similar analysis was again carried out to confirm the genotyping results. Identical results were obtained when the genotyping was performed on two separate occasions.

### 2.2. Statistical Analysis

Clinical characteristics of all the subjects were expressed as mean ± SD. The student’s t-test and chi-square test were used to analyse the variables. The frequencies of alleles were estimated by using the allele counting method and Hardy-Weinberg equilibrium assessment. A one-way ANOVA test was used to compare the group means. All statistical analyses were performed by using SPSS version 21.0 (SPSS Inc., Chicago, IL, USA); *p* < 0.05 was considered to be statistically significant.

## 3. Results

In this study, 80 ASD patients of Malay ethnicity were sampled. The control group, which are ASD patients without PAH, comprised of 50 (62.5%) samples, whereas 30 (37.5%) were ASD patients with PAH. A majority of the ASD patients with PAH were female (93.3%). Whereas for those diagnosed with ASD without PAH, 88% were females. Table 2 tabulates the clinical characteristics of subjects in both groups with respect to age, age at diagnosis, size of ASD, mean arterial pressure (MAP), and mPAP with *p* < 0.05.

**Figure 1:**
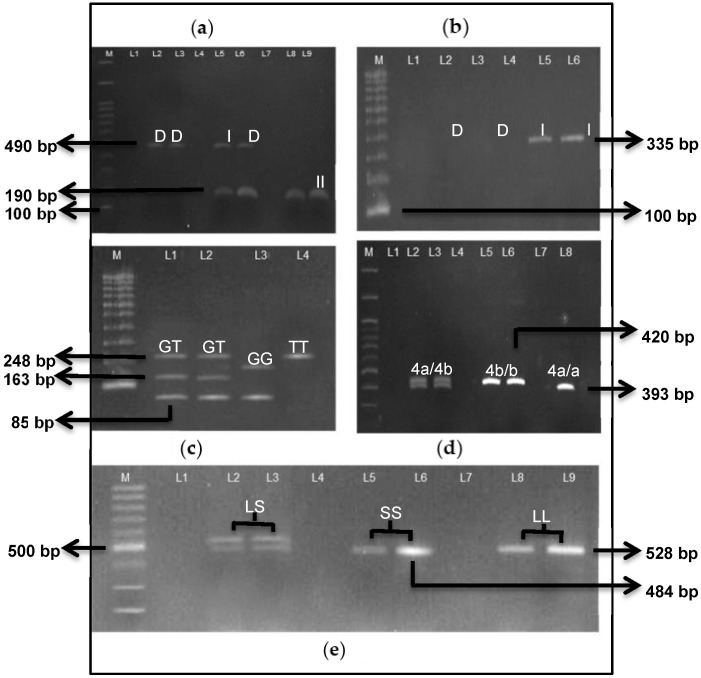
(**a**) Amplification of *ACE I/D* in 2% agarose gel. DD genotype was denoted by 490 bp band (Lane 2 & 3) and II genotype was shown by 190 bp band (Lane 8 & 9) whereas ID genotype showed both 190 bp and 490 bp bands (Lane 5 & 6). (**b**) Mistyping of *ACE I/D*. I allele showed 335 bp band (Lane 5 & 6) whereas DD genotype was proven by the absence of any band (Lane 2 & 3). (**c**) Restriction fragments in 4% agarose gel. Heterozygous type (GT genotype) showed three bands consists of 85 bp, 163 bp and 248 bp (Lane 1 & 2). GG genotype was showed by 85 bp and 163 bp bands (Lane 3). Lane 4 shows the undigested 248 bp band, which was TT genotype (mutant). (**d**) PCR product of *eNOS 4b/4a* amplification in 2% agarose. Heterozygous sample (4a/b) was shown by both 393 bp and 420 bp bands (Lane 2 & 3). Wild type (4b/b) was shown by a 420 bp band (Lane 5 & 6). 393 bp band indicated a mutant (4a/a) sample (Lane 8). (**e**) Amplification of *5-HTTLPR* in 1.5% agarose gel. A heterozygous type (LS genotype) showed both 484 bp and 528 bp bands (Lane 2 & 3). SS genotype was indicated by 484 b p band (Lane 5 & 6) whereas LL genotype was shown by a 528 bp band (Lane 8 & 9). M: 100-bp marker.

The mean age for the control subjects was higher than that of the case subjects. There was no significant difference observed for age and age patients diagnosed with ASD. However, patients with PAH were diagnosed to have ASD earlier than ASD patients who had not developed PAH. There was a significant difference found in the diameter of defects, MAP and mPAP. The size of ASD was found to be bigger in patients with PAH. Figure 1 shows the PCR products of each gene polymorphism, and Table 3 demonstrates the genotypic and allelic distributions of *ACE I/D*, *G894T* and *4b/4a* gene polymorphism of the *eNOS* gene and the *5-HTTLPR* gene polymorphism of the PAH and non-PAH groups.

There was no significant association found between the *ACE I/D* and both *eNOS G894T* and *eNOS 4b/4a* gene polymorphisms with the ASD subjects (*p* < 0.05). However, there is an association between the L allele of *5-HTTLPR* gene polymorphism with PAH among ASD patients (*p* < 0.05). A multivariate test (General Linear Model) was performed to identify the confounding factor for all the genetic polymorphisms, to compare the associations between genotypes and clinical parameters (Table 4). In this study, the clinical parameters analysed are age, diameter of defects, MAP, and mPAP. There were no significant differences found between the genotypes of all the gene polymorphisms of the genes studied and the risk factors in both groups.

## 4. Discussion

This study aims to identify the differences between ASD patients without PAH and ASD patients with PAH in terms of genetic makeup, as this may help us have more understanding on the pathophysiology of the disease. Candidate gene analysis enables the identification of the presence of known gene variations of candidate genes (*ACE*, *eNOS* and *5-HTTLPR*) that may be implicated in the pathogenesis of PAH. Thus, a genotype-phenotype association can be determined, and patients can then be classified into the high-risk group that may develop PAH, so that early therapeutic and preventive measures can be implemented. The findings from this study might give some new insight into the management of ASD patients.

A higher percentage of LL genotypes of *5-HTTLPR* with gene polymorphism was noticed in the PAH group. Our findings support the 5-HTT pathway in causing pulmonary vascular remodeling and PAH development by the overexpression of 5-HTT [20,21]. Patients with LL genotypes will express more than double the serotonin mRNA in the pulmonary artery muscle cells [22]. In this present study, we found that the L allele of the *5-HTTLPR* gene polymorphism was associated with an increased risk of possible PAH. To the best of our knowledge, this is the first molecular study on our population that investigates the association of the *5-HTTLPR* gene polymorphism with PAH in Malay ASD patients. It is in correspondence with studies by Eddahibi et al. (2001) [20] and Willers et al. (2006) [22], which suggested an association between the LL genotype and the risk of developing PH. Although statistical differences were not reached, higher mPAP was observed for the LL genotypes in the PAH group. These findings are in accordance with another study conducted on Spanish [22], Caucasian [23] and Turkish [24] subjects. Other than that, the diameter of defects were found to be larger for the SS and LS genotypes, but the mPAP was higher for the LL genotypes in the PAH group. It is suggested that patients who carry the LL genotype are more severe than those who carry the SS or LS genotype, despite the size of the defect, due to the higher level of mPAP in the LL group. Homozygous L alleles cause the double expression of serotonin in the primary pulmonary artery smooth muscle cells. This in turn leads to vascular smooth muscle hyperplasia and vascular remodeling, that cause an increase in the pulmonary artery pressure [20].

The deletion polymorphism of the *ACE* gene has been known to cause an increase in angiotensin II, and renin angiotensin system is involved in pulmonary artery remodeling [25]. However, different studies on the *ACE* DD genotype effect on cardiovascular diseases have produced different results [24,26]. Since there was no significant difference found in the *ACE* genotype distribution and allele frequencies among groups, it can be concluded that *ACE* gene polymorphisms may not be the risk factor in the development of PAH among Malay ASD patients. The result was similar to a study by Hoeper et al. (2003), in which there were no significant differences in mPAP between cases and control subjects with II, ID or DD genotypes [27].

A greater percentage of the G allele of *eNOS G894T* polymorphism in the non-PAH group (70.65%), rather that in the PAH patients (48.4%), was also observed in Toganel’s study [28]. In the present study, the incidence of G allele was almost similar between the control group (49%) and the AHD cases (48.33%). Another study by Vadapalli et al. (2010) [10] also showed that no significant difference was observed between idiopathic PAH and the control group. It is assumed that maybe there are gene-gene and gene-environment interactions that play a role in the function of *eNOS G894T* polymorphism regarding the pathogenesis of PAH in Malays.

In this present study, we found no association between the *eNOS 4b/4a* polymorphism and PAH in Malay ASD patients. The frequency of the “a” allele was higher in patients with PAH (23.33%) as compared to those with ASD (20%). However, these findings do not support past literature, which correlated a lower detection of NO metabolites with the presence of the “a” allele [29]. A higher percentage of the “b” allele was observed among the non-PAH group. This may suggest a protective effect of the “b” allele against PAH. Another study in Romania [28] on PAH among children with congenital heart diseases also gave negative results. The *eNOS 4b/4a* was also found to have no association with PH in COPD patients in both Turkish and Zurich populations [10,24].

Table 5 summarises the genotypic and allelic distributions of all the gene polymorphisms of the genes studied on several types of PAH in other populations, with contradictory results. This can be due to confounding factors, such as different ethnicities, or different environment backgrounds which have influences on the genetic makeup.

The present study has some limitations. Although our study sample was relatively small as compared to other epidemiological and association studies, the result of this study supports the hypothesis that the L allele of the *5-HTTLPR* gene has an association with PAH in ASD subjects. A more extensive study can be done in future with a larger sample size and more stringent inclusion and exclusion criteria to further confirm the hypothesis, and to confirm the association between the gene polymorphism of the *ACE*, *eNOS*, and *5-HTTLPR* genes. Further investigation is needed to understand the possible role of other polymorphisms of various genes in relation to ASD among the three ethnic races in Malaysia.

## 5. Conclusions

Among all the gene polymorphisms studied in this study, the *5-HTTLPR* (rs25531) can be considered as the candidate gene for the early development of PAH among Malay ASD patients.

## Figures and Tables

**Table 1:** Primers and PCR product for each gene polymorphisms.

Gene Polymorphisms	Primers	PCR Cycling Conditions		PCR Products (bp)	References
*ACE* I/D (rs4340)	Forward: 5′-CTG GAG ACC ACT CCC ATC CTT TCT-3′ Reverse: 5′-GAT GTG GCC ATC ACA TTC GTC ACG -AT-3′	Denaturation Annealing Extension Final Extension Number of Cycles	95 °C, 1 min 58 °C, 2 min 72 °C, 1 min 72 °C, 2 min 30 cycles	190—DD 490—II 40 & 190—ID	[20]
*ACE* mistyping	Forward: 5′-TGG GAC CAC AGC GCC CGC CAC TAC-3′ Reverse: 5′-TCG CCA GCC CTC CCA TGC CCA TAA-3′	Denaturation Annealing Extension Final Extension Number of Cycles	94 °C, 30 s 67 °C, 45 s 72 °C, 2 min 72 °C, 2 min 30 cycles	353—I allele no band—DD	[21]
*eNOS G894T* (rs1799983)	Forward: 5′-AAG GCA GGA GAC AGT GGA TGG A-3′ Reverse: 5′-CCC AGT CAA TCC CTT TGG TGC TCA-3′	Denaturation Annealing Extension Final Extension Number of Cycles	94 °C, 30 s 62.8 °C, 30 s 72 °C, 2 min 72 °C, 5 min 35 cycles	248	[22]
*eNOS 4b/4a*	Forward: 5′-AGG CCC TAT GGT AGT GCC TTT-3′ Reverse: 5′-TCT CTT AGT GCT GTG GTC AC-3′	Denaturation Annealing Extension Final Extension Number of Cycles	94 °C, 1 min 56 °C, 1 min 72 °C, 2 min 72 °C, 7 min 35 cycles	393—4a/a 420—4b/b 393 & 420—4a/b	[23]
*5-HTTLPR* (rs25531)	Forward: 5′-GGC GTT GCC GCT CTG AAT GC-3′ Reverse: 5′-GGC GTT GCC GCT CTG AAT GC-3′	Denaturation Annealing Extension Final Extension Number of Cycles	94 °C, 30 s 61 °C, 30 s 72 °C, 1 min 72 °C, 10 min 35 cycles	528—LL 484—SS 484 & 528—LS	[24]

**Table 2:** Clinical characteristics of PAH patients and control subjects.

Parameter	Case Subjects (ASD with PAH)	Control Subjects (ASD without PAH)	*p* Value
Gender, M/F	2/28	6/44	-
Age	33.33 ± 9.316	37.50 ± 14.971	0.174
Age diagnosed	28.97 ± 8.838	32.80 ± 14.474	0.194
Diameter of defects (cm)	3.19 ± 4.018	1.88 ± 0.852	* 0.028
MAP (mmHg)	89.30 ± 12.438	78.82 ± 18.169	* 0.007
mPAP (mmHg)	58.88 ± 14.53	22.04 ± 10.517	* 0.000

PAH, pulmonary arterial hypertension; ASD, atrial septal defect; MAP, mean arterial pressure; mPAP, mean pulmonary arterial pressure. * Significant *p* < 0.05. Values shown are mean ± SD.

**Table 3:** Distribution of genotypes and allele frequencies of each gene polymorphisms between PAH patients and control subjects.

Gene Polymorphism		PAH Patients (n = 30) n (%)	Control Subjects (n = 50) n (%)	*p* Value	Odds Ratio (95% Confidence Interval)
*ACE I/D*	Genotypes				0.68 (0.33–1.41)
	II	17 (56.67%)	22 (44%)	0.53	
	ID	12 (40%)	25 (50%)		
	DD	1 (3.33%)	3 (6%)		
	Alleles				
	I	46 (76.67%)	69 (69%)	0.30	
	D	14 (23.33%)	31 (31%)		
*eNOS G894T*	Genotypes				
	GG	0 (0%)	1 (2%)	0.63	0.98 (0.51–1.85)
	GT	29 (96.67%)	47 (94%)		
	TT	1 (3.33%)	2 (4%)		
	Alleles				
	G	29 (48.33%)	49 (49%)	0.94	
	T	31 (51.67%)	51 (51%)		
*eNOS 4b/4a*	Genotypes				
	4b/b (wild type)	21 (70%)	35 (70%)	0.57	1.217 (0.56–2.64)
	4a/a (mutant)	5 (16.7%)	5 (10%)		
	4a/b	4 (13.3%)	10 (20%)		
	Alleles				
	a	14 (23.33%)	20 (20%)	0.62	
	b	46 (76.67%)	80 (80%)		
*5-HTTLPR*	Genotypes				0.43 (0.22–0.82)
	SS	6 (20%)	23 (46%)	0.06	
	LL	12 (40%)	12 (24%)		
	LS	12 (40%)	15 (30%)		
	Alleles				
	S	24 (40%)	61 (61%)	* 0.01	
	L	36 (60%)	39 (39%)		

* Value (*p* < 0.05) was obtained using chi-square test as compared with controls. Data are reported as number of subjects, with percent in parentheses.

**Table 4:** Distribution between polymorphisms and clinical characteristics of the subjects.

Gene Polymorphism	ASD with PAH (Case)				ASD without PAH (Control)			
	Age *	Size of Defect *	MAP * (mmHg)	mPAP * (mmHg)	Age *	Size of Defect *	MAP * (mmHg)	mPAP * (mmHg)
*ACE I/D*								
II	32.71 ± 9.51	2.59 ± 0.88	90.71 ± 10.51	59.09 ± 17.53	36.95 ± 13.54	2.18 ± 0.77	72.86 ± 23.51	24.23 ± 12.59
ID	34.42 ± 9.75	4.29 ± 6.23	87.25 ± 15.48	58.92 ± 10.40	39.64 ± 16.03	1.66 ± 0.83	84.20 ± 10.79	20.64 ± 8.58
DD	31.00 ± 0.00	0.22 ± 0.00	90.00 ± 0.00	55.00 ± 0.00	23.67 ± 11.59	1.44 ± 1.24	77.67 ± 11.68	17.67 ± 7.64
*eNOS G894T*								
GG	-	-	-	-	29.00 ± 0.00	1.96 ± 0.00	77.00 ± 0.00	17.00 ± 0.00
GT	32.66 ± 8.69	3.22 ± 4.09	89.10 ± 12.61	58.98 ± 14.77	38.07 ± 15.48	1.91 ± 0.85	78.54 ± 18.90	35.11 ± 21.98
TT	53.00 ± 0.00	2.30 ± 0.00	95.00 ± 0.00	56.00 ± 0.00	31.67 ± 1.53	1.27 ± 0.90	83.67 ± 4.04	35.75 ± 17.21
*eNOS 4b/4a*								
4b/b	35.48 ± 9.82	3.60 ± 4.45	89.29 ± 13.17	58.07 ± 14.23	38.37 ± 16.24	1.94 ± 0.87	78.91 ± 11.78	21.69 ± 9.40
4a/b	27.50 ± 8.43	3.75 ± 3.59	93.25 ± 15.15	71.50 ± 13.48	36.90 ± 13.34	1.85 ± 0.82	78.50 ± 24.29	26.70 ± 14.47
4a/a	29.00 ± 3.54	1.05 ± 1.23	86.20 ± 7.40	52.20 ± 12.76	32.60 ± 8.17	1.47 ± 0.82	78.70 ± 5.40	15.20 ± 4.32
*5-HTTLPR*								
SS	36.00 ± 9.80	2.77 ± 0.77	93.33 ± 8.60	52.67 ± 11.38	38.52 ± 15.73	1.87 ± 0.87	78.35 ± 20.18	22.65 ± 11.27
LS	33.50 ± 10.71	4.57 ± 6.12	88.08 ± 11.57	59.08 ± 17.05	40.13 ± 14.82	1.98 ± 0.71	78.87 ± 19.70	22.20 ± 10.52
LL	31.83 ± 8.01	2.04 ± 1.09	88.50 ± 15.14	61.79 ± 13.28	32.25 ± 13.51	1.76 ± 1.02	79.66 ± 12.70	20.67 ± 11.45

Values are mean ± SD. * = not significant (*p* > 0.05).

**Table 5:** Genotypes and allele frequency distribution.

Population	Disease	No	Genotypes (%)				Allele (%)			Study
*ACE I/D*			II	ID	DD	*p* Value	I	D	*p* Value	
Malaysia	HPT	65	36.9	52.3	10.8	*	63.08	36.92	**	[14]
USA	PPH	60	45	35	20	**	-	-	-	[19]
Caucasian	COPD	66	22.73	46.97	30.3	*	-	-	-	[30]
Romania	PAH	29	6.90	58.62	34.48	*	39.58	60.42	*	[31]
German	PPH	51	9.8	58.82	31.37	NS	-	-	-	[27]
Iran	CAD	224	12.5	38.84	48.66	NS	31.92	68.08	NS	[32]
Malaysia	PAH	30	56.67	40.0	3.33	NS	76.67	23.33	NS	Current study
*eNOS G894T*			GG	GT	TT		G	T		
Germany	IPAH	16	27	53	20	NS	53	47	NS	[10]
India	IPAH	77	50.65	41.16	51.95	NS	72.73	27.27	NS	[11]
India	EH	226	139	82	5	***	79.65	20.35	***	[33]
Malaysia	PAH	30	0	96.67	3.33	NS	48.33	51.67	NS	Current study
*eNOS 4b/4a*			bb	ab	Aa	p	a	b		
Germany	IPAH	16	67	33	-	NS	83	17	NS	[10]
Turkey	PH in COPD	24	83	17	0	*	8.33	91.67	-	[25]
Swiss	PH in COPD	27	63	33	4	NS	20.37	79.63	-	[34]
Malaysia	ESRD	150	87.33	11.33	1.33	NS	93	7	NS	[35]
Malaysia	PAH	30	70	16.67	13.33	NS	23.33	58.78	NS	Current study
*5-HTTLPR*			SS	LS	LL	p	S	L		
Caucasian	IPAH	11	81.82	0	18.18	*	18.18	81.82	-	[9]
Germany	COPD + PAH	27	18.52	59.26	22.22	-	51.85	48.15	*	[10]
Spain	IPAH + 2° PAH	49	26.53	28.15	20.41	NS	46.94	53.06	-	[36]
Chinese	VSD-related PAH	140	50.71	36.43	12.86	*	31.07	68.93	-	[37]
Malaysia	PAH	30	20	40	40	NS	40	60	*	Current study

HPT, hypertension; PPH, primary pulmonary hypertension; COPD, cardiac obstructive pulmonary disease; PAH, pulmonary arterial hypertension; CAD, coronary artery disease; EH, essential hypertension; ESRD, end stage renal disease; IPAH, idiopathic pulmonary arterial hypertension; VSD, ventricular septal defect * (*p* < 0.05), ** (*p* < 0.01), *** (*p* < 0.001), at 5% level of significance, NS: not significant (*p* > 0.05).
